# [μ-*N*,*N*′-Bis(3-meth­oxy-2-oxidobenzyl­idene)propane-1,3-diamine]trinitratocopper(II)terbium(III) acetone solvate

**DOI:** 10.1107/S1600536808002080

**Published:** 2008-01-25

**Authors:** Liu Fei, Zhang Fang

**Affiliations:** a The College of Chemical Engineering & Materials, Eastern Liaoning University, 325 Wenhua Road, Yuanbao District, Dandong City, Liaoning Province 118003, People’s Republic of China

## Abstract

In the title complex, [CuTb(C_19_H_20_N_2_O_4_)(NO_3_)_3_]·CH_3_COCH_3_, the Cu^II^ atom is four-coordinated by two O atoms and two N atoms from the deprotonated Schiff base in a square-planar geometry, while the Tb^III^ atom is ten-coordin­ated by four O atoms from the deprotonated Schiff base and six O atoms from three bidentate nitrate anions. The compound is isostructural with the previously reported Gd^III^ analogue [Elmali & Elerman (2004 ▶). *Z. Naturforsch. Teil B*, **59**, 535–540], which was described in the space group *P*1 with two formula units in the asymmetric unit. The crystal stucture is, in fact, centrosymmetric and is described here in the space group *P* 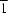 with one formula unit in the asymmetric unit.

## Related literature

For the isostructural Gd^III^ complex, see: Elmali & Elerman (2004 ▶). For a similar copper–cerium complex, see: Elmali & Elerman (2003 ▶).
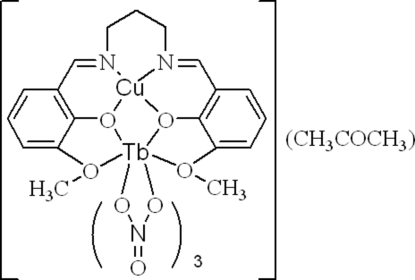


## Experimental

### 

#### Crystal data

[CuTb(C_19_H_20_N_2_O_4_)(NO_3_)_3_]·C_3_H_6_O
*M* *_r_* = 806.94
Triclinic, 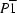

*a* = 9.388 (5) Å
*b* = 12.108 (6) Å
*c* = 13.604 (6) Å
α = 73.079 (16)°
β = 86.67 (2)°
γ = 72.33 (2)°
*V* = 1408.8 (12) Å^3^
*Z* = 2
Mo *K*α radiation
μ = 3.32 mm^−1^
*T* = 291 (2) K
0.19 × 0.16 × 0.14 mm

#### Data collection

Rigaku R-AXIS RAPID diffractometer
Absorption correction: multi-scan (*ABSCOR*; Higashi, 1995 ▶) *T*_min_ = 0.569, *T*_max_ = 0.659
12171 measured reflections
6275 independent reflections
5621 reflections with *I* > 2σ(*I*)
*R*_int_ = 0.025

#### Refinement

*R*[*F* ^2^ > 2σ(*F* ^2^)] = 0.025
*wR*(*F* ^2^) = 0.083
*S* = 1.11
6275 reflections
392 parameters
H-atom parameters constrained
Δρ_max_ = 0.74 e Å^−3^
Δρ_min_ = −0.50 e Å^−3^

### 

Data collection: *RAPID-AUTO* (Rigaku, 1998 ▶); cell refinement: *RAPID-AUTO*; data reduction: *CrystalStructure* (Rigaku/MSC, 2002 ▶); program(s) used to solve structure: *SHELXS97* (Sheldrick, 2008 ▶); program(s) used to refine structure: *SHELXL97* (Sheldrick, 2008 ▶); molecular graphics: *SHELXTL* (Sheldrick, 2008 ▶); software used to prepare material for publication: *SHELXL97*.

## Supplementary Material

Crystal structure: contains datablocks global, I. DOI: 10.1107/S1600536808002080/bi2275sup1.cif

Structure factors: contains datablocks I. DOI: 10.1107/S1600536808002080/bi2275Isup2.hkl

Additional supplementary materials:  crystallographic information; 3D view; checkCIF report

## Figures and Tables

**Table 1:** Selected bond lengths (Å)

Cu2—O1	1.939 (3)
Cu2—O3	1.947 (2)
Cu2—N2	1.957 (3)
Cu2—N1	1.989 (3)
O1—Tb1	2.352 (2)
O2—Tb1	2.506 (3)
O3—Tb1	2.344 (3)
O4—Tb1	2.492 (2)
O5—Tb1	2.470 (3)
O7—Tb1	2.501 (3)
O8—Tb1	2.455 (3)
O10—Tb1	2.494 (3)
O11—Tb1	2.491 (3)
O13—Tb1	2.564 (3)

**Fig. 1.**
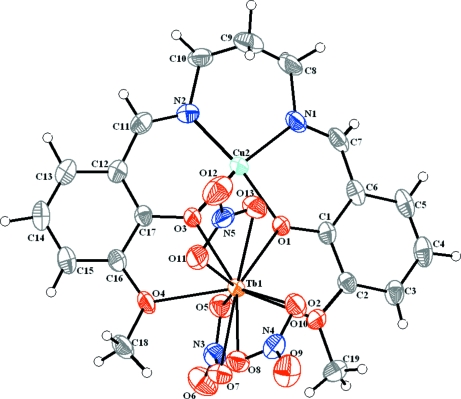
The molecular structure, showing 40% probability displacement ellipsoids for non-H atoms. The acetone solvent molecule is not shown.

## supplementary crystallographic information

### Comment

As shown in Fig. 1, the hexadentate Schiff base ligand links the Cu^II^ and Tb^II^ atoms into a dinuclear complex through two phenolate O atoms. The Tb^III^ atom is ten-coordinated by four O atoms from the ligand and six O atoms from three nitrate anions. The Cu^II^ atom is four-coordinated by two N atoms and two O atoms from the ligand. The acetone molecule is not associated with the complex. The complex is isostructural with its Gd^III^ analogue (Elmali & Elerman, 2004), although that was refined in space group *P*1 with two independent complexes in the asymmetric unit. A similar compound with Ce^III^ has also been reported (Elmali & Elerman, 2003).

### Experimental

The title complex was obtained by reaction of copper(II) acetate monohydrate (0.05 g, 0.25 mmol) with the Schiff base (0.0855 g, 0.25 mmol) in methanol/acetone (20 ml:5 ml). Terbium (III) nitrate hexahydrate (0.1126 g, 0.25 mmol) was added and the mixture was refluxed for 3 h. The mixture was then cooled and filtered, and diethyl ether was allowed to diffuse slowly into the filtrate. Single crystals were obtained after several days. Elemental analysis calculated: C 32.65, H 3.29, N 8.67; found: C 32.75, H 3.25, N 8.68.

### Refinement

H atoms bound to C atoms were placed in calculated positions and allowed to ride on their parent atoms, with C—H = 0.93 Å (C *sp*^2^), C—H = 0.97Å (methylene C), C—H = 0.96 Å (methyl C), and with *U*_iso_(H) = 1.2 or 1.5*U*_eq_(C).

### Crystal data

**Table:**

[CuTb(C_19_H_20_N_2_O_4_)(NO_3_)_3_]·C_3_H_6_O	*Z* = 2
*M**_r_* = 806.94	*F*_000_ = 798
Triclinic, *P*1	*D*_x_ = 1.902 Mg m^−^^3^
Hall symbol: -P 1	Mo *K*α radiation λ = 0.71073 Å
*a* = 9.388 (5) Å	Cell parameters from 11368 reflections
*b* = 12.108 (6) Å	θ = 3.2–27.5º
*c* = 13.604 (6) Å	µ = 3.32 mm^−^^1^
α = 73.079 (16)º	*T* = 291 (2) K
β = 86.67 (2)º	Block, green
γ = 72.33 (2)º	0.19 × 0.16 × 0.14 mm
*V* = 1408.8 (12) Å^3^	

### Data collection

**Table:**

Rigaku R-AXIS RAPID diffractometer	6275 independent reflections
Radiation source: fine-focus sealed tube	5621 reflections with *I* > 2σ(*I*)
Monochromator: graphite	*R*_int_ = 0.025
*T* = 291(2) K	θ_max_ = 27.5º
ω scans	θ_min_ = 3.1º
Absorption correction: multi-scan(ABSCOR; Higashi, 1995)	*h* = −12→12
*T*_min_ = 0.569, *T*_max_ = 0.659	*k* = −15→14
12171 measured reflections	*l* = −17→17

### Refinement

**Table:**

Refinement on *F*^2^	Secondary atom site location: difference Fourier map
Least-squares matrix: full	Hydrogen site location: inferred from neighbouring sites
*R*[*F*^2^ > 2σ(*F*^2^)] = 0.025	H-atom parameters constrained
*wR*(*F*^2^) = 0.083	*w* = 1/[σ^2^(*F*_o_^2^) + (0.0525*P*)^2^] where *P* = (*F*_o_^2^ + 2*F*_c_^2^)/3
*S* = 1.11	(Δ/σ)_max_ = 0.049
6275 reflections	Δρ_max_ = 0.74 e Å^−^^3^
392 parameters	Δρ_min_ = −0.50 e Å^−^^3^
Primary atom site location: structure-invariant direct methods	Extinction correction: none

### Special details

**Table:**

Geometry. All e.s.d.'s (except the e.s.d. in the dihedral angle between two l.s. planes) are estimated using the full covariance matrix. The cell e.s.d.'s are taken into account individually in the estimation of e.s.d.'s in distances, angles and torsion angles; correlations between e.s.d.'s in cell parameters are only used when they are defined by crystal symmetry. An approximate (isotropic) treatment of cell e.s.d.'s is used for estimating e.s.d.'s involving l.s. planes.
Refinement. Refinement of *F*^2^ against ALL reflections. The weighted *R*-factor *wR* and goodness of fit *S* are based on *F*^2^, conventional *R*-factors *R* are based on *F*, with *F* set to zero for negative *F*^2^. The threshold expression of *F*^2^ > σ(*F*^2^) is used only for calculating *R*-factors(gt) *etc*. and is not relevant to the choice of reflections for refinement. *R*-factors based on *F*^2^ are statistically about twice as large as those based on *F*, and *R*- factors based on ALL data will be even larger.

### Fractional atomic coordinates and isotropic or equivalent isotropic displacement parameters (Å^2^)

**Table:**

	*x*	*y*	*z*	*U*_iso_*/*U*_eq_	
C1	0.5484 (3)	0.7337 (3)	0.4160 (3)	0.0363 (7)	
C2	0.6382 (4)	0.7732 (3)	0.3361 (3)	0.0355 (7)	
C3	0.7913 (4)	0.7362 (3)	0.3494 (3)	0.0442 (8)	
H1	0.8502	0.7633	0.2961	0.053*	
C4	0.8589 (4)	0.6565 (4)	0.4446 (4)	0.0527 (10)	
H2	0.9624	0.6320	0.4537	0.063*	
C5	0.7741 (4)	0.6157 (3)	0.5227 (3)	0.0508 (10)	
H3	0.8202	0.5619	0.5842	0.061*	
C6	0.6164 (4)	0.6543 (3)	0.5113 (3)	0.0421 (8)	
C7	0.5320 (4)	0.6050 (3)	0.5939 (3)	0.0482 (9)	
H4	0.5879	0.5473	0.6503	0.058*	
C8	0.3378 (6)	0.5605 (5)	0.6968 (3)	0.0765 (15)	
H5	0.3421	0.5970	0.7508	0.092*	
H6	0.4050	0.4785	0.7172	0.092*	
C9	0.1832 (6)	0.5563 (4)	0.6869 (4)	0.0644 (12)	
H8	0.1638	0.4954	0.7459	0.077*	
H7	0.1742	0.5325	0.6259	0.077*	
C10	0.0688 (5)	0.6752 (4)	0.6798 (3)	0.0515 (9)	
H9	−0.0289	0.6632	0.6933	0.062*	
H10	0.0918	0.7081	0.7318	0.062*	
C11	−0.0654 (4)	0.8417 (3)	0.5466 (3)	0.0411 (7)	
H11	−0.1404	0.8382	0.5941	0.049*	
C12	−0.1088 (4)	0.9332 (3)	0.4497 (3)	0.0381 (7)	
C13	−0.2582 (4)	1.0089 (4)	0.4359 (3)	0.0467 (8)	
H12	−0.3229	1.0025	0.4903	0.056*	
C14	−0.3085 (4)	1.0922 (4)	0.3422 (3)	0.0517 (9)	
H13	−0.4075	1.1409	0.3338	0.062*	
C15	−0.2132 (4)	1.1044 (3)	0.2598 (3)	0.0445 (8)	
H14	−0.2484	1.1608	0.1968	0.053*	
C16	−0.0665 (4)	1.0325 (3)	0.2724 (2)	0.0350 (6)	
C17	−0.0123 (3)	0.9448 (3)	0.3682 (2)	0.0325 (6)	
C18	−0.0137 (5)	1.1106 (4)	0.0937 (3)	0.0528 (9)	
H15	−0.0872	1.0826	0.0705	0.079*	
H16	0.0692	1.1047	0.0487	0.079*	
H17	−0.0574	1.1934	0.0934	0.079*	
C19	0.6458 (5)	0.8876 (4)	0.1589 (3)	0.0559 (10)	
H18	0.7078	0.9296	0.1766	0.084*	
H19	0.5788	0.9410	0.1026	0.084*	
H20	0.7076	0.8187	0.1393	0.084*	
C20	0.1948 (9)	0.3899 (6)	−0.0076 (6)	0.105 (2)	
H24	0.1539	0.4022	−0.0743	0.158*	
H25	0.1212	0.3778	0.0431	0.158*	
H26	0.2814	0.3199	0.0078	0.158*	
C21	0.2373 (7)	0.4967 (4)	−0.0065 (4)	0.0723 (14)	
C22	0.3355 (8)	0.4820 (5)	0.0826 (5)	0.0892 (17)	
H21	0.3467	0.5590	0.0801	0.134*	
H22	0.4319	0.4262	0.0789	0.134*	
H23	0.2906	0.4515	0.1459	0.134*	
Cu2	0.24442 (4)	0.75725 (3)	0.49586 (3)	0.03510 (10)	
N1	0.3899 (4)	0.6295 (3)	0.6009 (2)	0.0473 (7)	
N2	0.0640 (3)	0.7635 (3)	0.5764 (2)	0.0388 (6)	
N3	0.3245 (4)	1.1092 (3)	0.2292 (3)	0.0481 (7)	
N4	0.3809 (3)	0.8115 (3)	0.0469 (3)	0.0479 (7)	
N5	0.1404 (4)	0.6928 (3)	0.2358 (3)	0.0500 (7)	
O1	0.4010 (3)	0.7715 (2)	0.39786 (18)	0.0432 (5)	
O2	0.5608 (3)	0.8481 (2)	0.24596 (19)	0.0437 (5)	
O3	0.1291 (2)	0.8745 (2)	0.37547 (17)	0.0392 (5)	
O4	0.0382 (3)	1.0369 (2)	0.19704 (18)	0.0426 (5)	
O5	0.3132 (3)	1.0309 (3)	0.3128 (2)	0.0533 (6)	
O6	0.3324 (4)	1.2069 (3)	0.2294 (3)	0.0737 (10)	
O7	0.3240 (3)	1.0788 (2)	0.1473 (2)	0.0492 (6)	
O8	0.3023 (3)	0.9175 (2)	0.0474 (2)	0.0493 (6)	
O9	0.4110 (4)	0.7857 (3)	−0.0338 (3)	0.0720 (9)	
O10	0.4244 (3)	0.7372 (2)	0.1352 (2)	0.0505 (6)	
O11	0.0991 (3)	0.7990 (2)	0.1769 (2)	0.0533 (6)	
O12	0.0819 (4)	0.6173 (3)	0.2310 (3)	0.0802 (11)	
O13	0.2473 (4)	0.6682 (2)	0.3006 (2)	0.0574 (7)	
O14	0.2026 (6)	0.5872 (4)	−0.0747 (3)	0.1106 (16)	
Tb1	0.285531 (15)	0.877969 (12)	0.234617 (10)	0.03350 (7)	

### Atomic displacement parameters (Å^2^)

**Table:**

	*U*^11^	*U*^22^	*U*^33^	*U*^12^	*U*^13^	*U*^23^
C1	0.0339 (15)	0.0339 (15)	0.0403 (17)	−0.0059 (12)	−0.0049 (13)	−0.0129 (13)
C2	0.0362 (15)	0.0292 (14)	0.0398 (17)	−0.0085 (12)	−0.0044 (13)	−0.0084 (13)
C3	0.0336 (16)	0.0386 (17)	0.063 (2)	−0.0112 (13)	−0.0018 (15)	−0.0174 (17)
C4	0.0353 (17)	0.051 (2)	0.072 (3)	−0.0046 (15)	−0.0165 (18)	−0.024 (2)
C5	0.046 (2)	0.0437 (19)	0.055 (2)	0.0031 (15)	−0.0219 (18)	−0.0149 (17)
C6	0.0411 (18)	0.0354 (16)	0.0444 (19)	0.0003 (13)	−0.0121 (15)	−0.0131 (15)
C7	0.056 (2)	0.0387 (18)	0.0319 (17)	0.0081 (15)	−0.0086 (16)	−0.0043 (14)
C8	0.077 (3)	0.066 (3)	0.040 (2)	0.009 (2)	0.010 (2)	0.022 (2)
C9	0.105 (4)	0.0377 (19)	0.048 (2)	−0.027 (2)	0.012 (2)	−0.0054 (17)
C10	0.057 (2)	0.060 (2)	0.0343 (18)	−0.0255 (19)	0.0065 (16)	−0.0008 (17)
C11	0.0421 (17)	0.0494 (19)	0.0397 (18)	−0.0193 (15)	0.0100 (14)	−0.0205 (16)
C12	0.0357 (16)	0.0404 (16)	0.0441 (18)	−0.0139 (13)	0.0031 (13)	−0.0187 (15)
C13	0.0377 (17)	0.057 (2)	0.053 (2)	−0.0160 (16)	0.0086 (15)	−0.0263 (18)
C14	0.0327 (17)	0.057 (2)	0.065 (2)	0.0007 (15)	−0.0070 (17)	−0.029 (2)
C15	0.0382 (17)	0.0423 (18)	0.051 (2)	−0.0019 (14)	−0.0113 (15)	−0.0180 (16)
C16	0.0363 (15)	0.0312 (14)	0.0363 (16)	−0.0056 (12)	−0.0046 (13)	−0.0118 (13)
C17	0.0304 (14)	0.0309 (14)	0.0371 (16)	−0.0090 (11)	−0.0020 (12)	−0.0107 (13)
C18	0.056 (2)	0.047 (2)	0.0378 (19)	0.0001 (17)	−0.0110 (17)	0.0009 (16)
C19	0.047 (2)	0.065 (2)	0.052 (2)	−0.0237 (19)	0.0100 (17)	−0.006 (2)
C20	0.127 (6)	0.070 (4)	0.099 (5)	−0.021 (4)	−0.041 (4)	0.004 (3)
C21	0.093 (4)	0.049 (2)	0.056 (3)	−0.006 (2)	0.018 (3)	−0.008 (2)
C22	0.124 (5)	0.057 (3)	0.080 (4)	−0.021 (3)	0.001 (4)	−0.017 (3)
Cu2	0.0369 (2)	0.03297 (19)	0.02827 (19)	−0.00746 (16)	0.00002 (15)	−0.00112 (16)
N1	0.0553 (18)	0.0390 (15)	0.0307 (14)	0.0011 (13)	0.0008 (13)	−0.0005 (12)
N2	0.0462 (15)	0.0427 (15)	0.0307 (14)	−0.0200 (13)	0.0034 (12)	−0.0085 (12)
N3	0.0469 (17)	0.0448 (17)	0.0512 (19)	−0.0138 (14)	−0.0015 (14)	−0.0113 (15)
N4	0.0419 (16)	0.0621 (19)	0.0450 (17)	−0.0191 (15)	0.0063 (13)	−0.0205 (16)
N5	0.0483 (17)	0.0406 (16)	0.060 (2)	−0.0183 (14)	0.0202 (15)	−0.0117 (15)
O1	0.0304 (11)	0.0507 (14)	0.0324 (11)	−0.0035 (10)	−0.0023 (9)	0.0036 (10)
O2	0.0343 (12)	0.0521 (14)	0.0374 (13)	−0.0126 (11)	0.0025 (10)	−0.0025 (11)
O3	0.0319 (11)	0.0414 (12)	0.0307 (11)	−0.0015 (9)	0.0007 (9)	0.0001 (10)
O4	0.0387 (12)	0.0368 (12)	0.0369 (12)	0.0031 (10)	−0.0076 (10)	−0.0011 (10)
O5	0.0615 (17)	0.0517 (15)	0.0443 (14)	−0.0160 (13)	0.0051 (12)	−0.0118 (13)
O6	0.095 (3)	0.0440 (16)	0.084 (2)	−0.0250 (16)	−0.011 (2)	−0.0140 (16)
O7	0.0602 (16)	0.0467 (14)	0.0375 (13)	−0.0200 (12)	0.0008 (12)	−0.0030 (11)
O8	0.0596 (16)	0.0458 (14)	0.0369 (13)	−0.0136 (12)	0.0043 (11)	−0.0065 (11)
O9	0.075 (2)	0.093 (2)	0.0543 (18)	−0.0167 (18)	0.0059 (16)	−0.0401 (18)
O10	0.0519 (15)	0.0448 (14)	0.0441 (14)	−0.0038 (11)	−0.0027 (12)	−0.0073 (12)
O11	0.0455 (14)	0.0491 (15)	0.0627 (17)	−0.0158 (12)	0.0011 (13)	−0.0104 (13)
O12	0.079 (2)	0.070 (2)	0.115 (3)	−0.0458 (19)	0.034 (2)	−0.042 (2)
O13	0.0661 (18)	0.0377 (13)	0.0569 (17)	−0.0112 (13)	0.0044 (14)	−0.0016 (12)
O14	0.157 (5)	0.065 (2)	0.077 (3)	−0.011 (3)	0.008 (3)	0.004 (2)
Tb1	0.03237 (9)	0.03267 (9)	0.02717 (9)	−0.00535 (6)	−0.00082 (6)	−0.00045 (6)

### Geometric parameters (Å, °)

**Table:**

C1—O1	1.332 (4)	C18—H16	0.960
C1—C2	1.399 (5)	C18—H17	0.960
C1—C6	1.418 (5)	C19—O2	1.435 (5)
C2—C3	1.375 (4)	C19—H18	0.960
C2—O2	1.382 (4)	C19—H19	0.960
C3—C4	1.417 (6)	C19—H20	0.960
C3—H1	0.930	C20—C21	1.469 (8)
C4—C5	1.357 (6)	C20—H24	0.960
C4—H2	0.930	C20—H25	0.960
C5—C6	1.414 (5)	C20—H26	0.960
C5—H3	0.930	C21—O14	1.181 (6)
C6—C7	1.428 (6)	C21—C22	1.502 (8)
C7—N1	1.279 (5)	C22—H21	0.960
C7—H4	0.930	C22—H22	0.960
C8—N1	1.476 (5)	C22—H23	0.960
C8—C9	1.483 (8)	Cu2—O1	1.939 (3)
C8—H5	0.970	Cu2—O3	1.947 (2)
C8—H6	0.970	Cu2—N2	1.957 (3)
C9—C10	1.493 (6)	Cu2—N1	1.989 (3)
C9—H8	0.970	Cu2—Tb1	3.4749 (16)
C9—H7	0.970	N3—O6	1.208 (4)
C10—N2	1.492 (5)	N3—O7	1.271 (4)
C10—H9	0.970	N3—O5	1.274 (4)
C10—H10	0.970	N4—O9	1.222 (4)
C11—N2	1.293 (5)	N4—O8	1.274 (4)
C11—C12	1.439 (5)	N4—O10	1.275 (4)
C11—H11	0.930	N5—O12	1.219 (4)
C12—C17	1.394 (5)	N5—O11	1.257 (4)
C12—C13	1.411 (5)	N5—O13	1.280 (5)
C13—C14	1.377 (6)	O1—Tb1	2.352 (2)
C13—H12	0.930	O2—Tb1	2.506 (3)
C14—C15	1.396 (6)	O3—Tb1	2.344 (3)
C14—H13	0.930	O4—Tb1	2.492 (2)
C15—C16	1.377 (5)	O5—Tb1	2.470 (3)
C15—H14	0.930	O7—Tb1	2.501 (3)
C16—O4	1.379 (4)	O8—Tb1	2.455 (3)
C16—C17	1.424 (4)	O10—Tb1	2.494 (3)
C17—O3	1.333 (4)	O11—Tb1	2.491 (3)
C18—O4	1.448 (4)	O13—Tb1	2.564 (3)
C18—H15	0.960		
			
O1—C1—C2	117.8 (3)	O1—Cu2—Tb1	40.14 (7)
O1—C1—C6	122.7 (3)	O3—Cu2—Tb1	39.95 (7)
C2—C1—C6	119.5 (3)	N2—Cu2—Tb1	130.52 (9)
C3—C2—O2	124.7 (3)	N1—Cu2—Tb1	129.85 (10)
C3—C2—C1	120.6 (3)	C7—N1—C8	115.4 (3)
O2—C2—C1	114.7 (3)	C7—N1—Cu2	123.8 (3)
C2—C3—C4	119.8 (4)	C8—N1—Cu2	120.7 (3)
C2—C3—H1	120.1	C11—N2—C10	115.0 (3)
C4—C3—H1	120.1	C11—N2—Cu2	124.3 (2)
C5—C4—C3	120.7 (3)	C10—N2—Cu2	120.7 (2)
C5—C4—H2	119.7	O6—N3—O7	123.1 (4)
C3—C4—H2	119.7	O6—N3—O5	120.8 (4)
C4—C5—C6	120.5 (3)	O7—N3—O5	116.1 (3)
C4—C5—H3	119.7	O9—N4—O8	121.0 (4)
C6—C5—H3	119.7	O9—N4—O10	123.8 (4)
C5—C6—C1	118.9 (4)	O8—N4—O10	115.2 (3)
C5—C6—C7	118.6 (3)	O12—N5—O11	121.5 (4)
C1—C6—C7	122.3 (3)	O12—N5—O13	122.0 (4)
N1—C7—C6	128.9 (3)	O11—N5—O13	116.5 (3)
N1—C7—H4	115.6	C1—O1—Cu2	128.2 (2)
C6—C7—H4	115.6	C1—O1—Tb1	124.0 (2)
N1—C8—C9	113.1 (4)	Cu2—O1—Tb1	107.76 (10)
N1—C8—H5	109.0	C2—O2—C19	118.0 (3)
C9—C8—H5	109.0	C2—O2—Tb1	118.0 (2)
N1—C8—H6	109.0	C19—O2—Tb1	123.2 (2)
C9—C8—H6	109.0	C17—O3—Cu2	129.3 (2)
H5—C8—H6	107.8	C17—O3—Tb1	122.90 (19)
C8—C9—C10	112.3 (4)	Cu2—O3—Tb1	107.83 (10)
C8—C9—H8	109.1	C16—O4—C18	117.7 (3)
C10—C9—H8	109.1	C16—O4—Tb1	117.60 (18)
C8—C9—H7	109.1	C18—O4—Tb1	122.5 (2)
C10—C9—H7	109.1	N3—O5—Tb1	96.8 (2)
H8—C9—H7	107.9	N3—O7—Tb1	95.4 (2)
N2—C10—C9	111.7 (3)	N4—O8—Tb1	97.5 (2)
N2—C10—H9	109.3	N4—O10—Tb1	95.6 (2)
C9—C10—H9	109.3	N5—O11—Tb1	98.5 (2)
N2—C10—H10	109.3	N5—O13—Tb1	94.4 (2)
C9—C10—H10	109.3	O3—Tb1—O1	63.45 (9)
H9—C10—H10	107.9	O3—Tb1—O8	146.77 (9)
N2—C11—C12	128.6 (3)	O1—Tb1—O8	147.69 (9)
N2—C11—H11	115.7	O3—Tb1—O5	72.83 (9)
C12—C11—H11	115.7	O1—Tb1—O5	73.37 (10)
C17—C12—C13	119.4 (3)	O8—Tb1—O5	118.67 (9)
C17—C12—C11	122.6 (3)	O3—Tb1—O11	81.12 (10)
C13—C12—C11	117.9 (3)	O1—Tb1—O11	116.73 (9)
C14—C13—C12	120.2 (4)	O8—Tb1—O11	72.71 (10)
C14—C13—H12	119.9	O5—Tb1—O11	143.71 (10)
C12—C13—H12	119.9	O3—Tb1—O4	65.89 (8)
C13—C14—C15	121.0 (3)	O1—Tb1—O4	126.51 (9)
C13—C14—H13	119.5	O8—Tb1—O4	85.75 (9)
C15—C14—H13	119.5	O5—Tb1—O4	76.54 (9)
C16—C15—C14	119.5 (3)	O11—Tb1—O4	69.87 (9)
C16—C15—H14	120.2	O3—Tb1—O10	138.29 (9)
C14—C15—H14	120.2	O1—Tb1—O10	99.56 (9)
C15—C16—O4	124.8 (3)	O8—Tb1—O10	51.53 (9)
C15—C16—C17	120.5 (3)	O5—Tb1—O10	142.00 (10)
O4—C16—C17	114.6 (3)	O11—Tb1—O10	73.34 (10)
O3—C17—C12	122.4 (3)	O4—Tb1—O10	130.23 (8)
O3—C17—C16	118.3 (3)	O3—Tb1—O7	115.07 (9)
C12—C17—C16	119.3 (3)	O1—Tb1—O7	117.69 (9)
O4—C18—H15	109.5	O8—Tb1—O7	67.18 (9)
O4—C18—H16	109.5	O5—Tb1—O7	51.50 (9)
H15—C18—H16	109.5	O11—Tb1—O7	124.64 (9)
O4—C18—H17	109.5	O4—Tb1—O7	70.61 (10)
H15—C18—H17	109.5	O10—Tb1—O7	106.57 (10)
H16—C18—H17	109.5	O3—Tb1—O2	124.00 (8)
O2—C19—H18	109.5	O1—Tb1—O2	64.58 (8)
O2—C19—H19	109.5	O8—Tb1—O2	89.00 (9)
H18—C19—H19	109.5	O5—Tb1—O2	73.82 (9)
O2—C19—H20	109.5	O11—Tb1—O2	142.46 (9)
H18—C19—H20	109.5	O4—Tb1—O2	142.70 (9)
H19—C19—H20	109.5	O10—Tb1—O2	69.68 (9)
C21—C20—H24	109.5	O7—Tb1—O2	73.33 (9)
C21—C20—H25	109.5	O3—Tb1—O13	71.12 (10)
H24—C20—H25	109.5	O1—Tb1—O13	68.46 (10)
C21—C20—H26	109.5	O8—Tb1—O13	105.29 (10)
H24—C20—H26	109.5	O5—Tb1—O13	136.01 (10)
H25—C20—H26	109.5	O11—Tb1—O13	50.51 (10)
O14—C21—C20	121.7 (6)	O4—Tb1—O13	109.79 (10)
O14—C21—C22	121.8 (5)	O10—Tb1—O13	67.18 (10)
C20—C21—C22	116.4 (5)	O7—Tb1—O13	172.48 (9)
C21—C22—H21	109.5	O2—Tb1—O13	107.22 (10)
C21—C22—H22	109.5	O3—Tb1—Cu2	32.23 (5)
H21—C22—H22	109.5	O1—Tb1—Cu2	32.10 (6)
C21—C22—H23	109.5	O8—Tb1—Cu2	165.72 (6)
H21—C22—H23	109.5	O5—Tb1—Cu2	75.60 (7)
H22—C22—H23	109.5	O11—Tb1—Cu2	95.49 (8)
O1—Cu2—O3	78.92 (10)	O4—Tb1—Cu2	97.89 (6)
O1—Cu2—N2	170.42 (11)	O10—Tb1—Cu2	118.11 (7)
O3—Cu2—N2	91.51 (12)	O7—Tb1—Cu2	127.06 (6)
O1—Cu2—N1	91.40 (12)	O2—Tb1—Cu2	96.16 (6)
O3—Cu2—N1	169.69 (12)	O13—Tb1—Cu2	60.46 (7)
N2—Cu2—N1	98.17 (13)		
